# Cases with IgG4-related ophthalmic disease with mass lesions surrounding the optic nerve

**DOI:** 10.1016/j.ajoc.2022.101324

**Published:** 2022-01-25

**Authors:** Shoko Hamaoka, Masayuki Takahira, Mitsuhiro Kawano, Kazunori Yamada, Dai Inoue, Tetsuhiko Okuda, Kazuhisa Sugiyama

**Affiliations:** a Department of Ophthalmology, Graduate School of Medical Science, Kanazawa University, Kanazawa, Japan; b Division of Rheumatology, Graduate School of Medical Science, Kanazawa University, Kanazawa, Japan; c Division of Radiology, Graduate School of Medical Science, Kanazawa University, Kanazawa, Japan; d Department of Hematology and Immunology, Graduate School of Medical Science, Kanazawa Medical University, Kanazawa, Japan

**Keywords:** IgG4-related disease, IgG4-related ophthalmic disease, Optic nerve, Optic neuropathy

## Abstract

**Purpose:**

IgG4-related ophthalmic disease (IgG4-ROD) is a lymphoproliferative disorder with representative symptoms including lacrimal gland enlargement (Mikulicz disease), masses around the trigeminal nerves, and extraocular muscle swelling. Herein, we describe cases of IgG4-ROD with lesions surrounding the optic nerve.

**Methods:**

Of the 56 consecutive patients (35 men and 21 women) with a “definite case” of IgG4-ROD diagnosed from November 2004 through December 2019 at Kanazawa University hospital, seven patients presented with mass lesions around the optic nerve based on magnetic resonance imaging, and four patients showed symptoms of optic neuropathy. The clinical courses of these seven cases were reviewed.

**Results:**

Among the 56 cases of IgG4-ROD, seven cases had lesions surrounding the optic nerve and all of these patients were male. The male dominance in the patient group with lesions surrounding the optic nerve was statistically significant based on a Chi-squared test (*p* < 0.001). Lacrimal gland swelling was also present in all seven cases, extraocular muscle enlargement in six cases, and trigeminal (infraorbital and supraorbital) nerve enlargement in six cases. Four patients showed deteriorated visual acuity compatible with optic neuropathy. These seven patients were treated by systemic steroid administration. Overall, in cases with optic neuropathy, visual function responded well to steroid therapy; however, recovery was limited in the worst case.

**Conclusions and importance:**

Attention should be paid for mass lesions surrounding the optic nerve in patients with IgG4-related disease, especially in cases with high serum IgG4 levels.

## 1 Introduction

IgG4-related disease (IgG4-RD) is a clinical entity that presents with serum IgG4 elevation and IgG4-positive staining of lymphoplasmacytic lesions in various systemic regions, such as the pancreas, lymph nodes, kidneys, lungs, and aorta.^1^^,^^2^ In the ophthalmic region, Mikulicz disease, which presents with symmetrical lacrimal gland swelling, was first identified as IgG4-RD in 2004.^3^ We were then among the first groups to report the detailed clinical and pathological manifestations of IgG4-related dacryoadenitis.^4^ According to the first international consensus for the nomenclature and clinical manifestations of IgG4-RD reported in 2012,^5^ representative ophthalmic lesions involve lacrimal gland enlargement and extraocular muscle swelling. Branches of the trigeminal nerves are also frequently involved.^6^ Subsequently, the triad of IgG4-related ophthalmic disease (IgG4-ROD) was established in the ophthalmic diagnostic criteria reported in 2015.^7^ An IgG4-RD disease registry in North America found ophthalmic involvement in 23% of 115 patients with IgG4-RD.^8^

Optic neuropathy may also occur in IgG4-ROD.^9^, ^10^, ^11^, ^12^, ^13^ However, there have only been sporadic case reports and little is known about its management and clinical course. In the present study, we describe cases of IgG4-ROD with lesions around the optic nerve, some presenting with optic neuropathy. We discuss the long term clinical courses and emphasize the importance of disease awareness, especially as a differential diagnosis for glaucoma.

## 2 Materials and methods

**Fig. 1:**
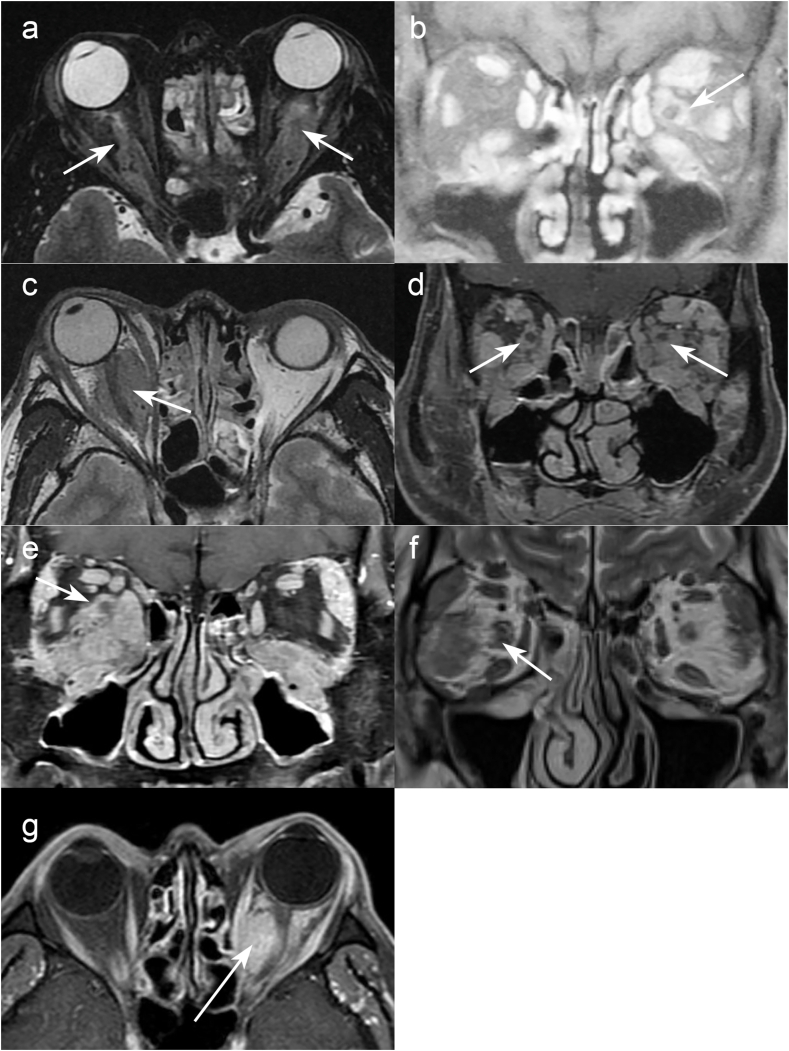
Magnetic resonance imaging (MRI) in cases with lesions surrounding the optic nerve and an orbital lesion pathology. Mass lesions around the optic nerves (arrows) were seen in seven cases of IgG4-related ophthalmic disease. All of these patients were male and the lesions were seen bilaterally around the optic nerve in case #1 (a: 52-year-old [y/o]) and case #4 (d: 57 y/o), around the left optic nerve in case #2 (b: 60 y/o) and case #7 (g: 69 y/o), and around the right optic nerve in case #3 (44 y/o), case #5 (e: 71 y/o) and case #6 (f: 44 y/o). Visual acuity was decreased due to optic neuropathy in four cases (#1, 2, 4, and 7).

From November 2004 through December 2019 at Kanazawa University hospital, 56 cases (35 males, 21 females) were diagnosed as a “definite case” of IgG4-ROD according to the diagnostic criteria established in 2015.^7^ Among these 56 consecutive cases, seven patients presented with mass lesions surrounding the optic nerve based on magnetic resonance imaging (MRI), and four cases showed symptoms of optic neuropathy. At diagnosis, the seven patients with IgG4-related optic nerve lesions were aged between 47 and 71 years old (median 65 years; Table 1) and all patients were male. In all of the 56 cases, IgH gene rearrangement was examined in the biopsied tissue and all were negative, excluding a diagnosis of lymphoma. Clinical presentations, treatments, and outcomes in these seven patients were investigated retrospectively. All patients were diagnosed as having IgG4-related ophthalmic disease based on the pathology of a lacrimal gland biopsy (case #1, 2, 3, 4, 5, 6), or an orbital mass lesion biopsy (case #7 in Table 1). The diagnostic criteria for IgG4-positive immunostaining of the biopsy samples was either a ratio of IgG4-positive cells to IgG-positive cells (IgG4+/IgG + cells) > 40% or a total number of IgG4-positive cells >50 per field of view under high-magnification microscopy.^7^ A mouse monoclonal anti-human IgG4 antibody (05-3800, Zymed, USA) and a rabbit polyclonal anti-human IgG antibody (A0423, Dako, USA) were used for immunostaining. Computed tomography (CT) and MRI were used to evaluate ophthalmic and other organ lesions at diagnosis and during steroid therapy. Oral steroid treatments were administered according to a protocol proposed by Masaki et al.^14^ Intravenous steroid treatment was adopted in one case with severe exophthalmos (case #3, Fig. 1c). Statistical analyses were performed by the Mann-Whitney *U* test and Chi-squared test using SPSS software (Version 24.0; SPSS Inc., Chicago, IL). For all analyses, *P* values less than 0.05 were considered statistically significant. This study adhered to the principles of the Declaration of Helsinki. Institutional Review Board (IRB)/Ethics Committee approval for this study was obtained from the Graduate School of Medical Sciences Kanazawa University.

**Table 1:** Clinical data from the seven patients with IgG4-related optic nerve lesions.

case#	age	sex	serum IgG4 (mg/dL) at diagnosis	orbital lesions			lesions surrounding optic nerve	optic neuropathy	pretreatment visual acuity right/left	visual acuity after steroid therapy	visual field defect	other organ involvement
				LG	EOM	TGN						
1	52	male	949	+	+	+	bilateral	bilateral	0.8/0.6	1.2/1.2	central scotoma	salivary gland, sinusitis
2	60	male	463	+	+	+	left	left	0.8/0.2	0.8/0.4	no data	salivary gland, dermatitis
3	44	male	599	+	–	+	right	–	1.5/1.5	1.5/1.5	none	none
4	67	male	2090	+	+	+	bilateral	bilateral	0.2/0.02	1.2/0.04	central scotoma (Fig. 2a)	lymph node, lung, liver, peri-aorta
5	71	male	2340	+	+	+	right	–	1.0/1.0	1.0/1.0	scotoma due to glaucoma	lung
6	65	male	2030	+	+	+	right	–	1.2/1.2	1.2/1.2	none	salivary gland, kidney
7	69	male	404	+	+	–	left	left	1.2/0.6	1.2/0.9	central scotoma (Fig. 2c)	none

LG: lacrimal glands, EOM: extraocular muscles, TGN: trigeminal nerves.

## 3 Results

Clinical data from the seven patients with IgG4-related optic nerve lesions are summarized in Table 1, listed in chronological order of their presentation at the clinic. All patients were male and four cases (# 1, 2, 4, and 7) were diagnosed with optic neuropathy, presenting with decreased visual acuity and visual field central scotoma. Serum IgG4 levels in these seven cases ranged from 404 to 2340 mg/dL (median: 949 mg/dL) before steroid therapy (Table 1), which was considerably elevated compared to the 135 mg/dL upper limit of the normal range of serum IgG4.

Representative views of the magnetic resonance images of the seven patients are shown Fig. 1. Other ophthalmic lesions included lacrimal gland swelling in all seven cases, extraocular muscle enlargement in six cases, and trigeminal nerve swelling in six patients.

There was one representative case without optic neuropathy but with a large unilateral mass surrounding the optic nerve (case #3) causing unilateral exophthalmos. However, the visual acuity of the affected eye was 1.2–1.5 throughout his clinical course. This patient underwent steroid pulse therapy and oral prednisolone, resulting in a reduction in the mass lesion.

In contrast, optic neuropathy was seen in four cases (#1, 2, 4, and 7) with lesions surrounding the optic nerve. Case #1 (52-year-old male) had a long history of IgG4-ROD with bilateral lacrimal gland swelling but optic nerve lesions were not initially detected. However, 7 years after the initial diagnosis of IgG4-related dacryoadenitis, lesions surrounding the optic nerves became apparent bilaterally, causing reduced visual acuity and bilateral central scotoma.

**Fig. 2:**
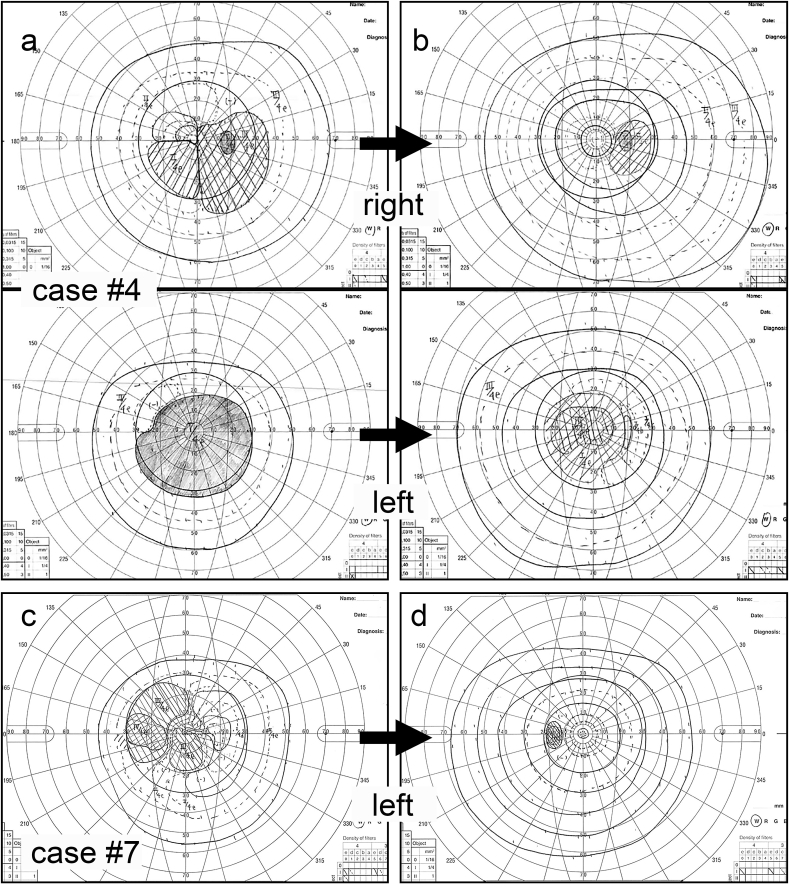
Visual field defects in patients with IgG4-related optic neuropathy. In case #4 (57-year-old [y/o] male) at initial examination, Goldmann perimetry showed bilateral central scotoma (a), which showed some improvement with oral steroid administration (b). Case #7 (69 y/o male) initially showed central scotoma in the left visual field (c), which was diminished within one month of oral steroid therapy (d).

The most representative case of IgG4-related optic neuropathy was case #4 (67-year-old male). At the initial visit to our hospital his visual acuity was 0.5/0.03 in the right/left eyes and IOP was around 10 mmHg; the patient was prescribed glaucoma eyedrops under the diagnosis of normal tension glaucoma at previous eye clinic for years. However, repeated examination of the visual field indicated optic neuropathy rather than glaucoma (Fig. 2a). Subsequent orbital MRI showed mass lesions around the optic nerves and enlargement of the lacrimal glands, trigeminal nerves, and extraocular muscles, suggestive of IgG4-related ophthalmic disease (Fig. 1d). His serum IgG4 level was extremely high at 2090 mg/dL. A lacrimal gland biopsy diagnosed IgG4-related dacryoadenitis without lymphoma. Whole body CT presented multiple systemic lesions consistent with IgG4-related disease, such as in the pituitary gland, lung, mediastinal lymph nodes, gastric antrum, liver, abdominal aorta, and retroperitoneum. The patient underwent oral prednisolone therapy tapering from 30 mg/day. Following steroid treatment his visual field (Fig. 2b) and visual acuity improved to 1.2/0.04 in the right/left eyes within one month; this is compared to the lowest values of 0.2/0.02 prior to treatment.

Another case with IgG4-related optic neuropathy (case #7, male) did not show other systemic lesions outside the orbit. This patient presented with an orbital mass around the left optic nerve, reduced left visual acuity of 0.6 and visual field scotoma as shown in Fig. 2c. Subsequent oral prednisolone therapy improved visual acuity to 0.9 and diminished the central scotoma within one month of treatment (Fig. 2d).

**Fig. 3:**
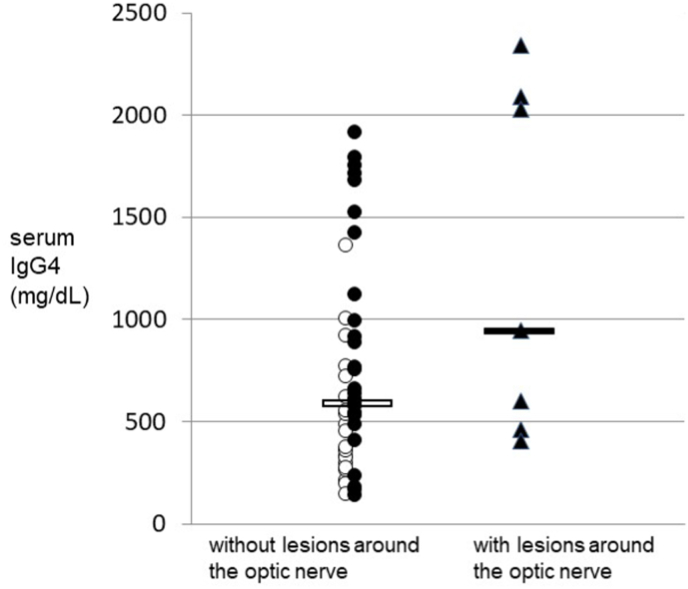
Serum IgG4 levels before treatment in cases with (closed circles, n = 7) or without (open circles, n = 49) lesions around the optic nerve. All patients were diagnosed with IgG4-ROD from November 2004 through December 2019 at Kanazawa University hospital. Serum IgG4 levels in cases without optic nerve lesions (median 575 mg/dL, range 136–1910 mg/dL) are shown as open circles (female: n = 21) and closed circles (male: n = 21). Only males showed optic nerve lesions and their serum IgG4 levels (median 949 mg/dL, range 463–2340 mg/dL) are shown as closed triangles (n = 7). It is noteworthy that the three cases with the highest serum IgG4 levels (greater than 2000 mg/dL) all belonged to the optic nerve lesion group; however, there was no statistical significance between the groups with or without optic nerve lesions. Horizontal bars indicate the median values.

Prior to steroid treatment, the serum IgG4 levels in these seven patients with optic nerve lesions were compared with those of 49 other IgG4-ROD cases without optic nerve lesions (Fig. 3). Interestingly, among both groups, the three cases with serum IgG4 levels greater than 2000 mg/dL were all males that showed optic nerve lesions. However, average serum IgG4 levels among the optic nerve lesion group (median 949 mg/dL, range 404–2340 mg/dL) were not significantly different (*P* = 0.074 by the Mann-Whitney *U* test) from the group without optic nerve lesions (median 575 mg/dL, range 136–1750 mg/dL). The complete male dominance in the patient group with lesions surrounding the optic nerve was statistically significant based on a Chi-squared test (*P* < 0.001). Compared to female patients, the serum IgG4 levels in male patients were significantly greater (*P* = 0.003 using the Mann-Whitney *U* test).

## 4 Discussion

The characteristic triad of IgG4-related ophthalmic disease includes swelling of the lacrimal glands, trigeminal nerves and extraocular muscles,^6^^,^^7^ whereas the complication of optic neuropathy seems to be rare and little is known about its incidence to date. In the literature, only sporadic cases of IgG4-related optic neuropathy have been reported. Soussan et al.^9^ reported a case of a 55-year-old woman with an IgG4-related optic nerve lesion accompanied by multiple paravertebral masses, but detailed ophthalmic findings were unreported. Kubota et al.^10^ presented MRI data from a 60-year-old male patient with compressive optic neuropathy and an orbital apex lesion, but no further information regarding treatment or outcomes was described. Takahashi et al.^11^ recently reported a case of a 62-year-old man with bilateral optic neuropathy responding to an oral steroid, but did not provide actual data of the visual field defects. Chen et al.^12^ reported a 78-year-old male case of severe compressive optic neuropathy due to IgG4-related orbital myositis managed by orbital decompression with adjunctive rituximab; however, the clinical course of visual field deterioration was not described. Plaza et al.^13^ also reported a case of optic neuropathy in a 37-year-old man among their case series of 11 patients with IgG4-related ophthalmic disease. Other case reports described IgG4-positive lesions around the optic nerves,^15^^,^^16^ while the serum IgG4 level was within normal level^15^ or not measured.^16^

It is very interesting that in the present study all cases with lesions surrounding the optic nerve were males. We saw one case of IgG4-related optic neuropathy in a female, but she did not present with lesions around the optic nerve; instead, the optic nerve was compressed by the swollen extraocular muscles at the orbital apex. As described above, most previous cases reported as optic neuropathy due to lesions surrounding the optic nerve were male patients^10,11,15,16.^ Sogabe et al.^17^ recently reported the location and frequency of lesions in an imaging study of 65 patients with IgG4-related ophthalmic disease. Frequent lesions included the triad of lacrimal glands (88%), trigeminal nerves (39%) and extraocular muscles (25%), whereas six patients (9%) presented with impaired visual acuity, visual field defects, or both due to optic nerve neuropathy. Interestingly, among these six cases of optic neuropathy, two cases were female, but neither of them presented with a mass around the optic nerve.

It is noteworthy that IgG4-related optic neuropathy may be misdiagnosed or attributed to glaucoma. For example, case #4 was originally managed by another clinic as normal tension glaucoma for years, whereas MRI at our hospital revealed optic neuropathy caused by mass lesions surrounding the optic nerve. In addition, case #1 initially showed an elevation in bilateral intraocular pressure around 25–30 mmHg, and underwent topical glaucoma medication with a diagnosis of steroid-induced ocular hypertension. However, seven years later, he showed visual filed defect accompanied by the lesions around the optic nerve in MRI, suggesting IgG4-related optic neuropathy.

The data from our case series showed that impaired visual acuity and visual field defects in IgG4-related optic neuropathy can respond well to steroid treatment and recover as shown in case #7 (Fig. 2c and d). However, in some cases recovery was not complete and some damage remained. One representative patient (case #4) with a long history of treatment for glaucoma, deterioration of left visual acuity as well as scotoma, did not recover sufficiently with steroid therapy. Thus, early stage diagnosis of IgG4-related optic neuropathy may be important for preserving visual function.

This study has several limitations that should be noted. First, the number of cases is small and the incidence of lesions surrounding the optic nerve among IgG4-ROD patients (7/56) could be quite different in a larger study population. Second, this is a retrospective study using medical records. Therefore, a prospective investigation across multiple institutes would be desirable for further elucidation of the pathology and epidemiology of IgG4-related optic neuropathy.

## 5 Conclusions

Attention should be paid to locations of orbital lesions and visual functions in patients with IgG4-related disease, especially in cases presenting high serum IgG4 levels. Visual function defects due to IgG4-related optic neuropathy could be reversible to some extent by oral steroid therapy.

## Patient consent

This study adhered to the principles of the Declaration of Helsinki and the HIPPA compliance statement. Institutional Review Board (IRB)/Ethics Committee approval for this study was obtained from the Graduate School of Medical Sciences Kanazawa University.

Patient consent was obtained in every case.

## Funding

This work was supported by Health and Labour Sciences Research Grants for the Study of Intractable Diseases from the Ministry of Health, Labour and Welfare, Japan.

The following authors have no financial disclosures: SH, MT, TO, MK, KY, KY, DI, TO, KS.

## Authorship

All authors attest that they meet the current ICMJe criteria for Authorship.

## Declaration of competing interest

None.
